# Natural Killer Cells Promote Kidney Graft Rejection Independently of Cyclosporine A Therapy

**DOI:** 10.3389/fimmu.2019.02279

**Published:** 2019-09-24

**Authors:** Muhammad Imtiaz Ashraf, Attia Sarwar, Anja A. Kühl, Elena Hunger, Arne Sattler, Felix Aigner, Heinz Regele, Martina Sauter, Karin Klingel, Stefan Schneeberger, Thomas Resch, Katja Kotsch

**Affiliations:** ^1^Department of Surgery, Charité-Universitätsmedizin Berlin, Berlin, Germany; ^2^Department of General, Visceral and Vascular Surgery, Charité-Universitätsmedizin Berlin, Berlin, Germany; ^3^iPath.Berlin—Immunopathology for Experimental Models, Berlin Institute of Health (BIH), Charité-Universitätsmedizin Berlin, Berlin, Germany; ^4^Clinical Institute of Pathology, Medical University of Vienna, Vienna, Austria; ^5^Department of Molecular Pathology, Tübingen University Hospital, Tübingen, Germany; ^6^Department of Visceral, Transplant and Thoracic Surgery, Center of Operative Medicine, Medical University of Innsbruck, Innsbruck, Austria

**Keywords:** kidney transplantation, Natural Killer (NK) cells, cyclosporine A, graft rejection, immunosuppression

## Abstract

Natural Killer (NK) cells have recently been recognized as key players in antibody-mediated chronic allograft failure, thus requiring a comprehensive understanding whether NK cells can escape conventional immunosuppressive regimens. Influence of cyclosporine A (CyA) on NK cell function was studied in a mouse model of allogeneic kidney transplantation (KTX, BALB/c to C57BL/6). Recipients were treated daily with CyA (10 mg/kg) for seven or 14 days for long term survival (day 56). Administration of CyA in recipients resulted in significantly reduced frequencies of intragraft and splenic CD8^+^ T cells, whereas the latter illustrated reduced IFNγ production. In contrast, intragraft and splenic NK cell frequencies remained unaffected in CyA recipients and IFNγ production and degranulation of NK cells were not reduced as compared with controls. Depletion of NK cells in combination with CyA resulted in an improvement in kidney function until day 7 and prolonged graft survival until day 56 as compared to untreated controls. Surviving animals demonstrated higher intragraft frequencies of proliferating CD4^+^FoxP3^+^Ki67^+^ regulatory T (T_REG_) cells as well as higher frequencies of CD8^+^CD122^+^ T_REG_. We here demonstrate that NK cell depletion combined with CyA synergistically improves graft function and prolongs graft survival, suggesting that NK cell targeting constitutes a novel approach for improving KTX outcomes.

## Introduction

Following kidney transplantation (KTX), commonly used immunosuppressive regimens are prevalently based on initial calcineurin inhibitors (CNIs), either cyclosporine A (CyA), or tacrolimus (Tac) (1). Following this approach, the incidence of acute T cell-mediated rejection has been effectively reduced, resulting in 1-year graft survival rates between 88 and 95% for renal allografts (2). However, CNIs primarily target T cells, whereas their efficacy in suppressing other immune subsets—such as Natural Killer (NK) cells—might be limited (3, 4).

While it is well known that NK cells are the primary effector cells of rejection after MHC-mismatched bone marrow (BM) transplantation (5, 6), until recently the role of NK cells in solid organ transplantation (SOT) has been widely underrated (7). However, increasing evidence now substantiates the notion that NK cells are involved in several facets of graft rejection, also after SOT, although their exact function remains controversial, suggesting a dichotomous role in transplantation (8). Whereas, inactivation of CD28-mediated co-stimulation of T cells failed to achieve acceptance of allogeneic vascularized cardiac grafts, additional inhibition of NK cells resulted in long-term allograft survival (9).

A potential contribution of NK cells to graft rejection was suggested by the observation that NK1.1^+^ cells provide help for alloantigen-specific T cells (10). Especially with respect to kidney injury, NK cells can recognize and kill tubular epithelial cells based on NKG2D ligand expression being upregulated in response to ischemia-reperfusion injury (IRI) (11). Despite the fact that these observations underline a pro-inflammatory role of NK cells, contrarily also pro-tolerogenic properties of NK cells in the context of SOT have been observed by killing donor antigen-presenting cells (12). More importantly, evidence of NK cells and their role in antibody-mediated rejection of kidney grafts is accumulating, as it was recently demonstrated that antibody-mediated rejection (ABMR) associates with an intrarenal expression signature enriched with NK cell pathways (13, 14).

However, data addressing the effect of CyA on NK cells are mostly drawn from peripheral blood NK cells as well as from *in vitro* studies and remain conflicting (15–19). Therefore, this study aims to delineate the effects of CyA on NK cells for the first time in a murine model of KTX in order to define the influence of NK cells on renal allograft outcome *in vivo*.

## Methods

### Animals

BALB/c and C57BL/6 mice were purchased from Charles River Laboratories (Charles River, Sulzfeld, Germany). Male mice weighing between 24 and 30 g were used for all experiments. Animals were housed under standard conditions and received humane care in compliance with the “Principles of Laboratory Animal Care” prepared by the National Academy of Sciences and published by the National Institutes of Health (NIH Publication No. 86–23, revised 1985). All animal experiments were approved by the Landesamt für Gesundheit und Soziales Berlin (G0089/16) or by the Austrian Federal Ministry of Science and Research (BMWF-66.011/0163-II/3b/2012).

### Kidney Transplantation

Allogeneic renal transplantations were performed as previously described (20). Briefly, the left donor kidney was flushed *in situ* with histidine-tryptophane-ketoglutarate solution (Custodiol®, Dr. Franz Köhler Chemie GmbH, Bensheim, Germany) and procured. End-to-side anastomoses between the donor renal vessels and the recipient's abdominal aorta and inferior vena cava were performed following a knotless technique (21). For urinary tract reconstruction the ureter was directly anastomosed into the bladder. The duration of cold and warm ischemia of allografts was maintained at 30 min. each. The contralateral native kidney was removed 24 h before sacrificing the animal on post operative day (POD) 7. For long-term surviving animals the contralateral kidney was removed on POD7 and the surviving animals were sacrificed on POD56. Animals with surgical complications were excluded from the study.

### *In vivo* Treatment

Beginning on the day of KTX (POD0), CyA was administered to C57BL/6 recipients until POD7 using daily subcutaneous injections at dosages of 10 mg/kg body weight. However, to prevent acute rejection in the long survival groups, recipients were treated daily with CyA for 14 days, as previously described (22). Depletion of NK cells *in vivo* was performed by intraperitoneal injection of an anti-mouse NK1.1 monoclonal antibody (200 μg; PK136, BioXCell, Lebanon, NH, USA) on POD−2 and POD+2.

### *In vitro* Assays

Functional analysis of NK and T cells was performed as recently described by using isolated splenic mononuclear cells (MNC) (23). Cells were stimulated in the presence of 200 U/ml mIL-2 either with 50 ng phorbol 12-myristate 13-acetate (PMA), 1 μg ionomycin calcium salt (Sigma-Aldrich, St. Louis, MO, USA), alternatively with murine YAC-1 cells as target cells (with an effector:target ratio 2:1), 5 μg/ml brefeldin A (Sigma Aldrich), and 2 μM monesin (Biolegend) for 4 h at 37°C and 5% CO_2_. After stimulation, cells were stained with antibodies listed in Supplemental Table 1. Degranulation capacity was assessed by CD107a lysosome-associated membrane protein-1 (LAMP-1) expression. Cell activation was assessed by first fixing and permeabilizing cells using Transcription Factor Staining Buffer Set (eBioscience) and then by staining intracellularly for IFNγ.

### Flow Cytometry

MNCs from spleen and lymph nodes were isolated by Ficoll-Histopaque (Sigma Aldrich) density gradient centrifugation. To obtain single cell suspension from kidneys the tissue was digested with collagenase IV (Gibco/Invitrogen, Darmstadt, Germany) plus DNase (Ambion/Applied Biosystems, Darmstadt, Germany) in 10 ml of supplemented RPMI for 45 min at 37°C. Released leukocytes were first separated by passing through a cell strainer (100 μm) and leukocytes were enriched using CD45 MicroBeads (Miltenyi Biotec, Inc., Auburn, CA, USA). For flow cytometry, 1 × 10^6^ cells were incubated for 20 min at 4°C for surface stainings and 30 min at room temperature for intracellular stainings with respective antibodies as listed in Supplemental Table 1. Cells were analyzed on a FACSFortessaX20 (BD Bioscience, Heidelberg, Germany), collecting a total of 100.000 events in a live gate, and data were analyzed using FlowJo software 10.0 (Tree Star Inc., Ashland, OR, USA). An exemplary gating strategy is provided in Supplemental Figure 1.

### Measurement of Antibody Concentrations

Antibody concentrations were assessed in recipient serum by a flow cytometry bead-based analysis applying a mouse immunoglobulin isotyping panel (LEGENDPlex Multi-Analyte Flow Assay Kit, Biolegend) according to the manufacturer's instructions and measured on a FACSFortessaX20 (BD Bioscience).

### Histology and Immunohistology

For immunohistochemistry, 5 μm paraffin tissue sections were deparaffinized and incubated for 1 h at 25°C with monoclonal rabbit anti-CD3 antibody (clone SP7, Thermo Scientific, Waltham, MA, USA) and with polyclonal goat anti-mouse C3d antibody (R&D Systems, Minneapolis, MN, USA). Controls using normal sera were run to exclude non-specific staining. Slides were processed using the Promark rabbit-on-rodent HRP Polymer Kit and the Promark goat-on-rodent HRP Polymer Kit (Biocare Medical, Concord CA, USA) followed by HistoGreen (Linaris, Wertheim, Germany) as substrate and counterstained with hematoxylin (24). In order to evaluate histomorphology, 1–2 μm kidney sections were cut, dewaxed, and histochemically stained with hematoxylin and eosin (H&E), periodic acid-Schiff (PAS), and the Elastica van Gieson staining kit (Merck) for connective tissue. Histological lesions were scored according to the definition of standard BANFF Classification (25).

### Serum Analysis of Kidney Function Parameters

Serum samples were stored in aliquots at −20°C until serum creatinine and urea were measured using the CREP2 Creatinine Plus version 2 and Urea/BUN assays, respectively, on a Roche/Hitachi Cobas C 701/702 system (Roche Diagnostics, Mannheim, Germany).

### Real-Time RT-PCR

Real-time RT-PCR was performed as recently described (24). In brief, total RNA from snap-frozen biopsies was extracted using the RNeasy Mini Kit (Qiagen, Hilden, Germany) according to the manufacturer's instructions. Integrity of RNA was checked using a NanoDrop™ 2000c spectrophotometer. For cDNA synthesis, 2 μg of total RNA was reverse transcribed in a 40 μl reaction volume using oligo(dT) primer and the RevertAidTM H Minus M-MuLV Reverse Transcriptase (Fermentas GmbH, St. Leon-Rot, Germany). Samples were tested for genomic DNA contamination and if tested positive excluded from the study. Real-time reverse transcription polymerase chain reaction (RT-PCR) for gene expression analysis was performed with the ABI PRISM 7500 Sequence Detection System (Life Technologies, Carlsbad, CA, USA). Primers were directly purchased as Taqman® gene expression assays (Life Technologies, Carlsbad, CA, USA) (Supplemental Table 2). Specific gene expression was normalized to the housekeeping gene hypoxanthine-guanine phosphoribosyltransferase (HPRT) using the formula 2–ΔCt. The mean Ct values for the genes of interest and the housekeeping gene were calculated from double determinations. Samples were considered negative if the Ct values exceeded 40 cycles.

### Statistics

Statistical analysis was performed using GraphPad Prism 6 (GraphPad Software, La Jolla, CA, USA). Kaplan-Meier plots were used to analyze graft survival, and the log-rank test was applied to assess the statistical significance of differences between survival curves. Statistical significance between two groups was calculated using the Mann-Whitney *U*-test. For multiple comparisons, Kruskal-Wallis test with Dunn's *post-hoc* was used. Statistical significance was considered for the following *p*-values: ns = *p* > 0.05, *p ≤ 0.05, **p ≤ 0.01.

## Results

### CyA Treatment Modulates Leukocyte Frequencies in Graft, Spleen, and Lymph Nodes

**Figure 1:**
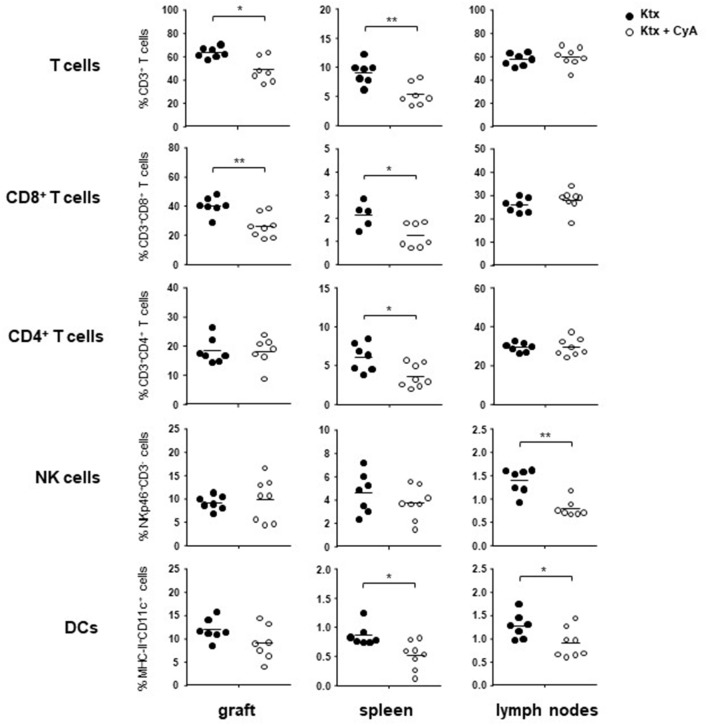
Administration of CyA leads to modulation of intragraft lymphocyte frequencies. After CyA administration, C57BL/6 recipient mice of BALB/c kidneys showed on POD7 significantly decreased frequencies of T cells in the allograft as well as in the spleen as compared with untreated recipients. NK cell frequencies were solely affected as a consequence of CyA treatment in the lymph nodes, whereas DC frequencies were significantly decreased in both spleen and lymph nodes, but not in the graft. Data are presented as mean of *n* = 7–8 animals/group. Cells were pre-gated on live CD45^+^ events. Statistically significant differences between naïve and CyA-treated animals were tested with the Mann-Whitney *U*-test; *p < 0.05, **p < 0.01.

In order to determine whether treatment with CyA results in a modification of the major lymphocyte frequencies *in vivo*, we sacrificed C57BL/6 recipients of BALB/c kidneys on postoperative day (POD) 7. Compared to untreated controls, T cells were significantly reduced in grafts as well as in the spleens of CyA-treated recipients. Especially frequencies of CD8^+^ T cells were reduced in both organs, whereas a significant reduction in CD4^+^ T cells was detected solely for spleens. Contrarily, CyA treatment resulted in significantly reduced frequencies of CD3^−^NKp46^+^ NK cells in lymph nodes and CD11c^+^MHCII^+^ dendritic cells (DCs) in both spleen and lymph nodes (Figure 1). Focusing on T cell subsets, we detected reduced frequencies of CD8^+^ CD44^+^CD62L^−^ effector memory T cells (T_EM_) in all investigated organs derived from CyA-treated animals. A reduction in CD4^+^CD44^+^CD62L^−^ T_EM_ cells was detected solely in lymph nodes (Supplemental Figure 2).

### *In vivo* Treatment With CyA Reduces NKG2D^+^ NK Cell Frequencies

**Figure 2:**
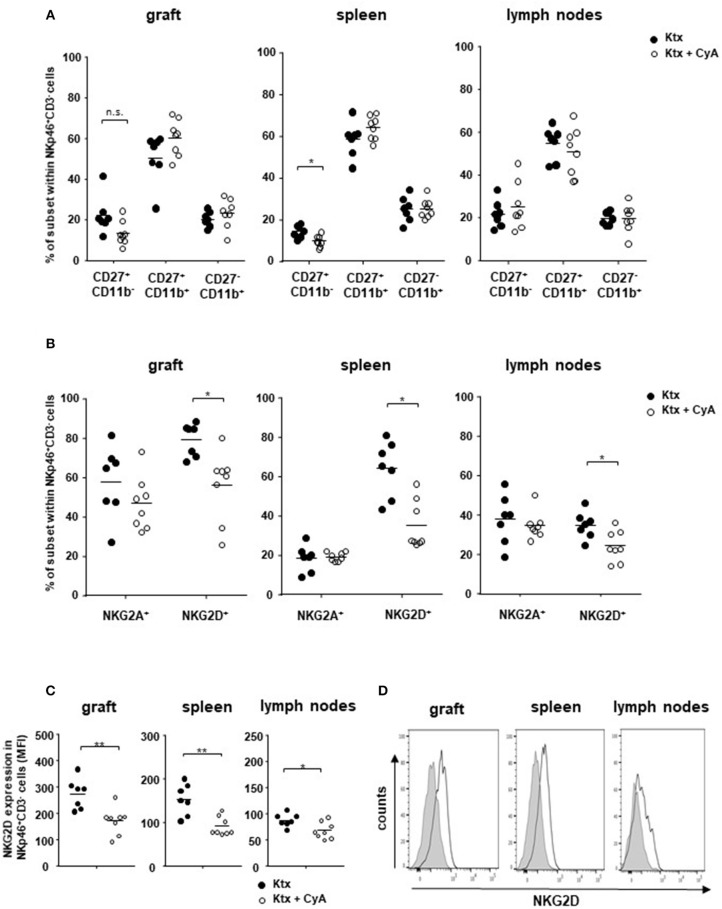
Impact of CyA on NK cell subsets. **(A)** CD3^−^NKp46^+^ NK cell subsets were analyzed according to their CD11b and CD27 expression. An influence of CyA treatment was detected for immature CD11b^−^CD27^+^ cells in the spleen. **(B)** CyA leads to a significant reduction in NKG2D^+^ NK cells in all three investigated organs, whereas the NKG2A^+^ NK cell subset remains unaffected. **(C,D)** NKG2D is significantly down-regulated on NK cells as a consequence of CyA, as reflected by mean fluorescence intensity (MFI). Data are presented as mean of *n* = 7–8 animals/group. Cells were pre-gated on live CD45^+^ events. Statistically significant differences between naïve and CyA-treated animals were tested with the Mann-Whitney *U*-test; *p < 0.05. Black line corresponds to NKp46^+^ NK cells derived from untreated animals, gray area to NK cells derived from CyA-treated animals. **p < 0.01.

Although we could not observe changes in the graft for bulk NK cell frequencies (Figure 1), we detected slightly reduced frequencies of CD27^+^CD11b^−^ immature NK cells in the kidney transplant as well as significantly reduced levels in the spleen derived from CyA animals, whereas no changes were observed for mature CD27^+^CD11b^+^ NK cells (Figure 2A). Recently, we reported on the functional importance of the activating Natural killer group 2 member D (NKG2D) C-type lectin receptor for allograft survival (23). In contrast to NK cells bearing the inhibitory NKG2A, frequencies of NK cells expressing the cytotoxicity receptor NKG2D were significantly decreased in all three investigated organs derived from the CyA group (Figure 2B). This observation was further underlined by the significantly decreased surface expression levels of NKG2D, as reflected by their mean fluorescence intensity, on NK cells (Figures 2C,D). Contrarily, CD3^+^ NKG2D^+^ T cell frequencies and surface expression levels of NKG2D on T cells were not affected by CyA treatment (Supplemental Figure 3).

### CyA Decreases IFNγ Production of T Cells but Not of NK Cells

**Figure 3:**
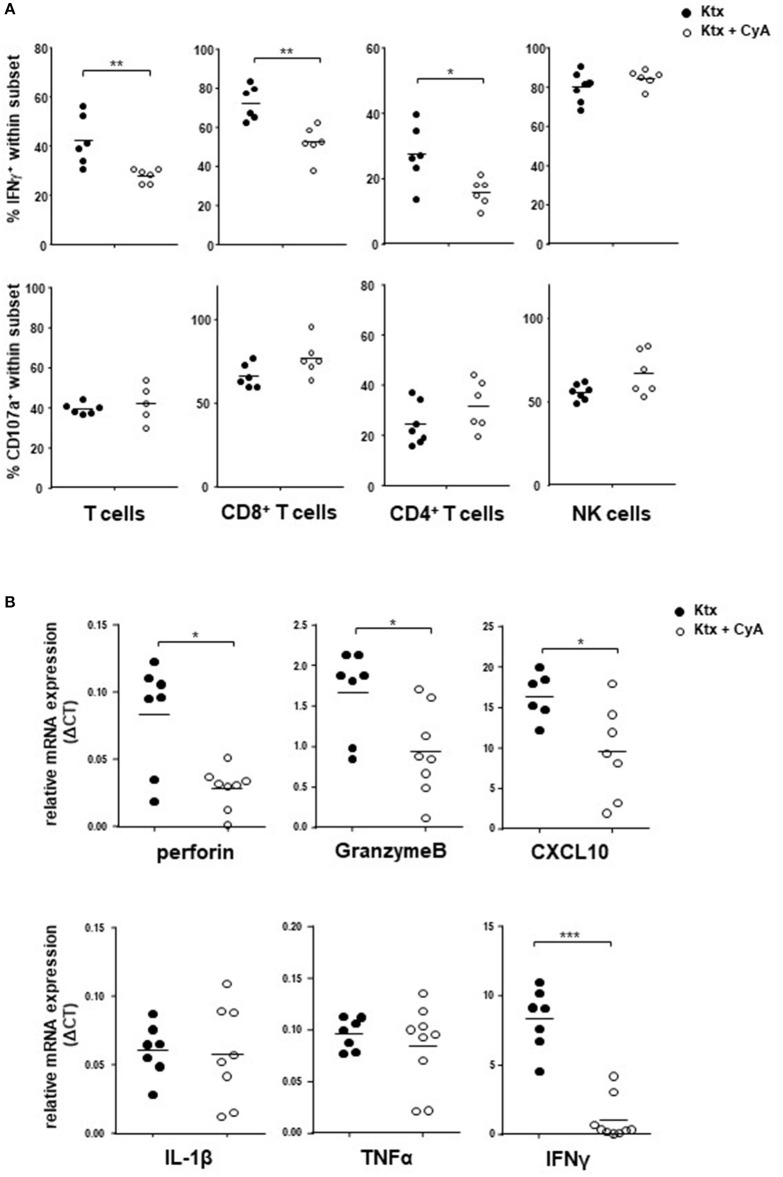
IFNγ production of NK cells is unaffected in CyA-treated recipients. **(A)** Splenocytes derived from CyA animals or recipients left untreated were polyclonally stimulated with PMA and ionomycin. Whereas both CD3^+^CD4^+^ and CD3^+^CD8^+^ T cells derived from CyA animals illustrate a clear reduction in IFNγ, CD3^−^NKp46^+^ NK cells retain their capacity to produce IFNγ. No impact was observed for the degranulation capacity. **(B)** Assessment of the intragraft mRNA profile revealed a significant reduction in inflammatory mediators including perforin, granzyme B, CXCL10, and IFNγ in CyA-treated recipients. Data are presented as mean of *n* = 7–8 animals/group. Cells were pre-gated on live CD45^+^ events. Statistically significant differences between naïve and CyA-treated animals were tested with the Mann-Whitney *U*-test; *p < 0.05, **p < 0.01, ***p < 0.001.

In order to examine whether CyA affects T and NK cell function *in vivo*, we isolated spleen cells from CyA or untreated recipients on POD7. Whereas, both CD4^+^ T cells and CD8^+^ T cells isolated from CyA-treated animals illustrated a significant reduction in their potential to secrete IFNγ as compared to that of untreated animals, IFNγ production by NK cells was not affected. No influence of CyA treatment was observed on the degranulation capacity of NK and T cells (Figure 3A). Using the murine cell line YAC-1 as target cells, a significant reduction of IFNγ was observed for CD8^+^ T cells, however no differences for degranulation and IFNγ production of NK cells derived from CyA treated animals compared with naïve recipients (Supplemental Figure 4).

Analysis of intragraft mRNA expression revealed that CyA kidneys showed significantly reduced mRNA levels of the cytotoxic markers perforin and granzyme B (*p* < 0.05). Moreover, mRNA expression of the chemokine *C-X-C motif chemokine 10* (CXCL10) and of IFNγ was also significantly reduced in grafts derived from the CyA group (Figure 3B), whereas no differences were observed for the inflammatory cytokines IL-1ß and TNFα.

### CyA in Combination With NK Cell Depletion Does Not Improve Kidney Function in the Short Term

**Figure 4:**
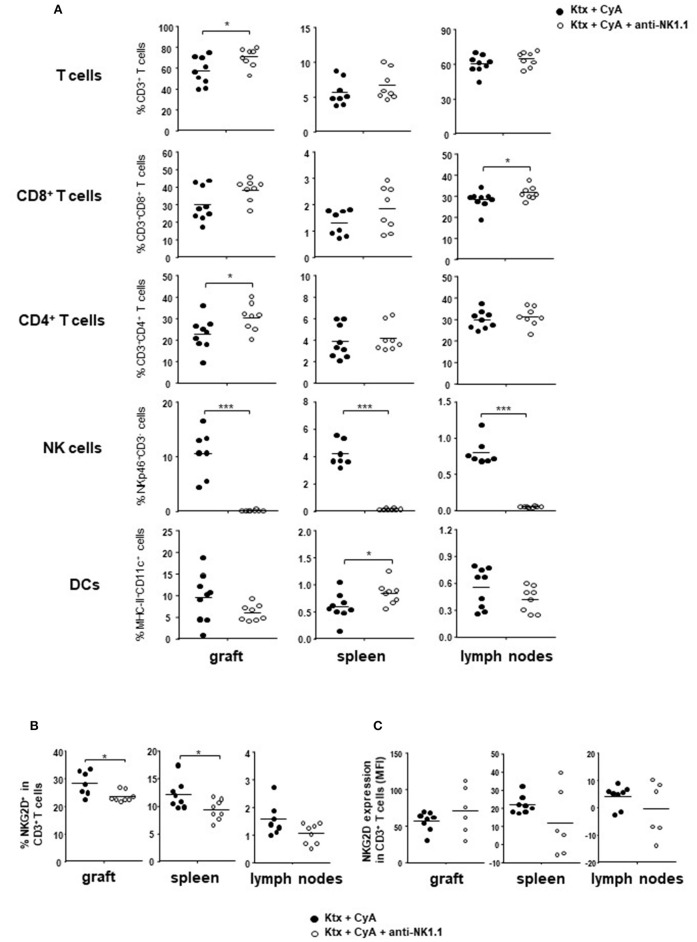
Short-course depletion of NK cells in combination with CyA increases intragraft CD4^+^ T cells. **(A)** NK depletion at POD-2 and POD+2 results in increased intragraft T cell frequencies at POD7, whereas NK cells were efficiently depleted in the kidney, spleen and lymph nodes. **(B)** Frequencies of NKG2D^+^CD3^+^ T cells are significantly reduced in graft and spleen following NK cell depletion, whereas **(C)** NKG2D is not downregulated on T cells as reflected by mean fluorescence intensity (MFI). Data are presented as mean of *n* = 7–8 animals/group. Cells were pre-gated on live CD45^+^ events. Statistically significant differences between naïve and CyA-treated animals were tested with the Mann-Whitney *U*-test; *p < 0.05, **p < 0.01, ***p < 0.001.

Our results illustrated so far suggest that NK cells in the recipient are insufficiently targeted by CyA treatment. In order to evaluate whether the additional NK cell neutralization results in improved graft survival, we depleted NK cells in CyA-treated C57BL/6 recipients of BALB/c kidneys on day POD-2 as well as on POD+2. The two-fold application of NK1.1 antibody resulted in efficient depletion of NK cells in the graft, spleen, and lymph nodes (Figure 4A, Supplemental Figure 5). Compared with graft recipients receiving CyA only, analysis on POD7 revealed a significant increase in CD4^+^ T cells in the allograft, whereas CD8^+^ T cells were increased in lymph nodes (Figure 4A). More interestingly, overall frequencies of CD3^+^NKG2D^+^ T cells were significantly reduced in the allograft as well as in the spleen (Figure 4B), whereas NKG2D expression levels were not affected on T cells (Figure 4C).

**Figure 5:**
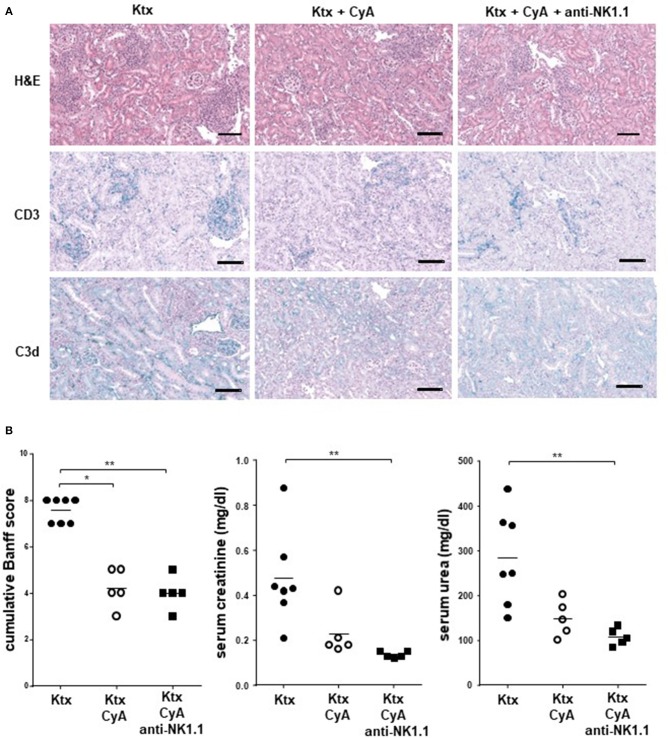
CyA in combination with NK cell depletion results in improved kidney function. **(A)** Sections of kidney grafts on POD were stained with hematoxylin and eosin (H&E), CD3 and C3d deposition. Histological analysis of renal allografts illustrates a massive cell lymphocyte infiltration in untreated animals. The photographs are representative of 7–8 animals in each group, magnification × 250 (scale bar = 100 μm) **(B)** Compared with the untreated group, a reduced BANFF Score, serum creatinine (mg/dl) and serum urea (mg/dl) were detected in the CyA as well as in the CyA+NK cell group. Data are presented as mean of *n* = 5–7 animals/group. Statistically significant differences between experimental groups were tested applying the Kruskal-Wallis test with Dunn's *post-hoc* test; *p < 0.05, **p < 0.01.

**Figure 6:**
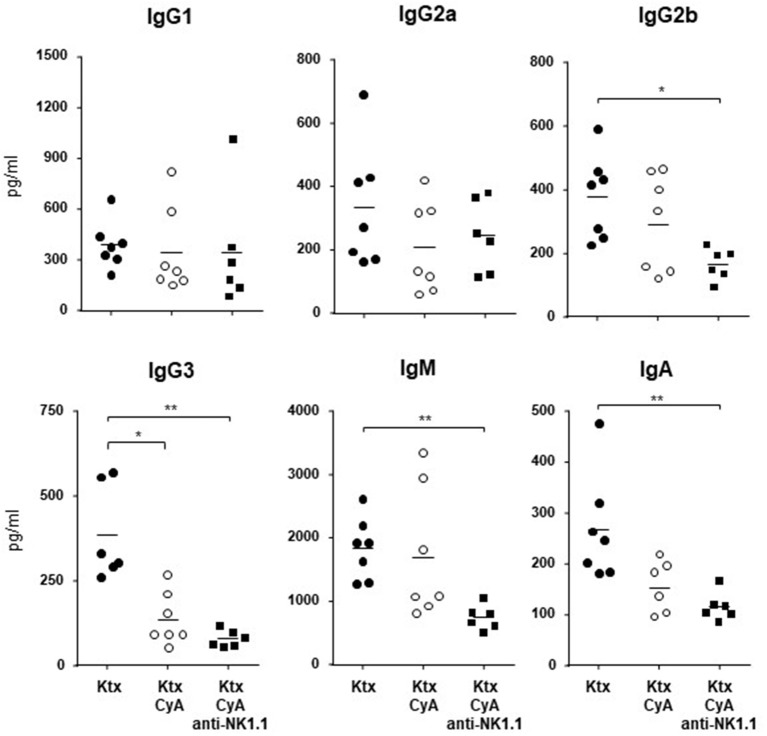
Antibody concentrations of kidney graft recipients. NK cell depletion in combination with CyA results in significantly lower frequencies of IgG2b, IgG3, IgM, and IgA as compared with naïve kidney recipients. No difference was detected for the CyA vs. the CyA+NK cell group. Data are presented as mean of *n* = 6–7 animals/group. Statistically significant differences between experimental groups were tested applying the Kruskal-Wallis test with Dunn's *post-hoc* test; *p < 0.05, **p < 0.01.

Although CyA treatment of the recipient resulted in decreased frequencies of graft-infiltrating lymphocytes as compared with untreated animals, this was not further improved by NK cell depletion. A similar picture was observed for CD3^+^ T cells, whereas a diffuse C3d deposition was detected for all groups (Figure 5A). However, the CyA group demonstrated an improved Banff Score compared to the untreated group, an observation which was not improved by combined CyA+NK treatment. In contrast, kidney function based on urea levels and creatinine were significantly improved in the CyA+NK group compared to the untreated group (Figure 5B). As NK cells are currently discussed as important contributors to humoral rejection post-kidney transplantation (13, 14), we therefore assessed the antibody response in the short-term surviving animals. CyA treatment of the recipient resulted in a clear decrease in IgG3 antibody concentrations, whereas the additional depletion of NK cells resulted in a significant decrease in the majority of antibody subclasses as compared with graft recipients left untreated (*p* < 0.05, *p* < 0.01, respectively) (Figure 6).

### CyA in Combination With NK Cell Depletion Improves Allograft Survival in the Long Term

**Figure 7:**
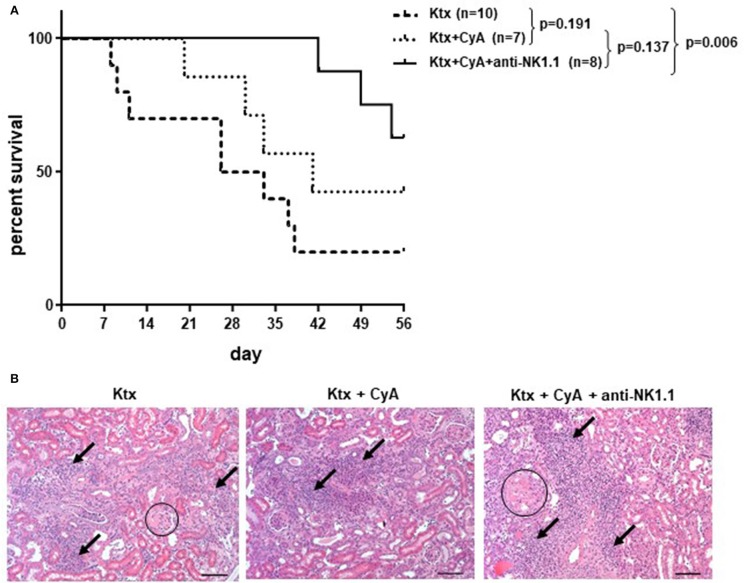
CyA and NK cell depletion prolongs renal allograft survival. **(A)** Compared with recipients left untreated, NK cell depletion in combination with CyA resulted in significantly improved long-term survival of kidney grafts until POD56. Contrarily, recipients receiving CyA did not demonstrate significantly improved graft survival as compared with naïve control recipients (median survival 41 vs. 29.5 days). The survival data were analyzed by Log-rank test and are presented as Kaplan-Meier survival curve. **(B)** H&E stained kidney sections showing sclerotic glomeruli (circle) and interstitial inflammation (arrow) (original magnification × 250; scale bar = 100 μm).

Although our findings in the short-term survival groups did not indicate a superior morphological benefit of NK cell depletion in combination with CyA treatment, the additional depletion of NK cells resulted in a significant overall survival until POD56 as compared with the control group (Figure 7A). However, histological scoring revealed no significant differences among the surviving animals in the experimental groups (Figure 7B, Table 1).

**Table 1:** Histopathological evaluation of renal allografts in the mice surviving until POD56 using standard Banff Classification (median).

**Histological lesions**	**KTX** **(*n* = 2)**	**KTX + CyA** **(*n* = 3)**	**KTX + CyA+anti-NK1.1** **(*n* = 5)**
Interstitial inflammation	3	3	2
Tubulitis	3	3	3
Glomerulitis	1.5	1	2
Arteritis	0.5	0	0
Capillaritis	2	3	2
Fibrosis	0	0	0
Tubular atrophy	2	3	2
Glomerulopathy	0.5	1	1
Mesangial matrix increase	0	0	0
Intimal fibrous thickening	0	0	0
Total	12.5	14	12

*Banff Score (0–3; 0 = Nil, 1 = below 25%, 2 = 25–50%, 3 = above 50%)*.

**Figure 8:**
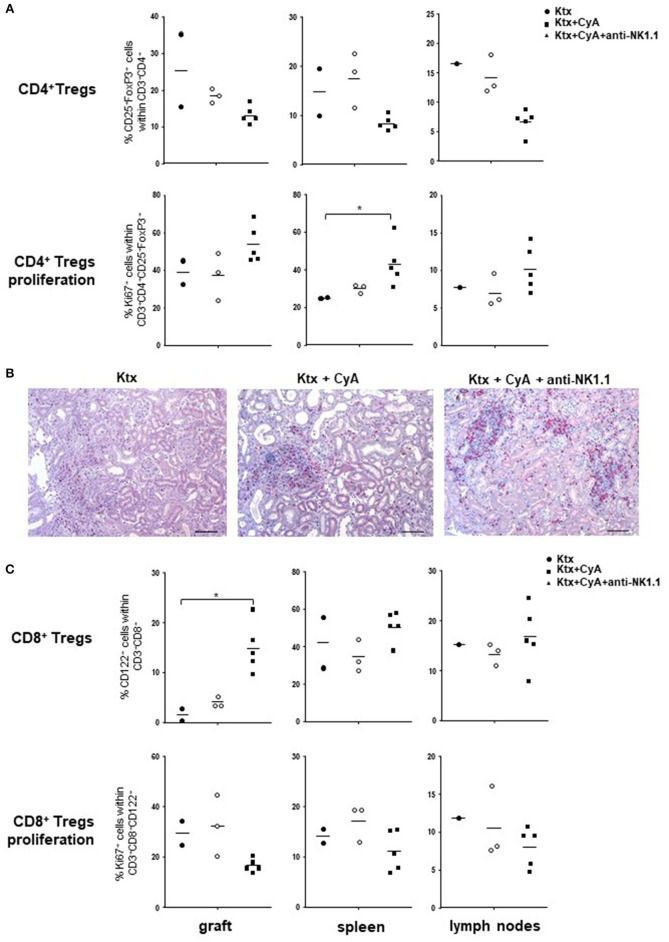
CyA in combination with NK cell depletion increases T_REG._ **(A)** Compared with recipients treated with CyA only, lower frequencies of CD4^+^ T_REG_ were detected in grafts, spleen and lymph nodes when NK cells were depleted in the early operative period. CD4^+^ T_REG_ were highly activated as reflected by Ki67 expression. **(B)** CD4^+^ (red) Foxp3^+^ (brown) T_REG_ cells were diffusely distributed in the interstitial infiltrate within the graft. Original magnification × 250 (scale bar = 100 μm). **(C)** In contrast to CD4^+^ T_REG_, intragraft CD8^+^CD122^+^ were increased, but demonstrate decreased proliferation. Data are presented as scatter dot plot with mean of surviving animals (*n* = 2–5)/group. Cells were pre-gated on live CD45^+^ events. Statistically significant differences between experimental groups were tested applying the Kruskal-Wallis test with Dunn's *post-hoc* test; *p < 0.05.

In order to explore mechanisms involved in the improved graft survival in the CyA+NK group, we analyzed animals that survived until POD56 for their regulatory T cell (T_REG_) composition. Intriguingly, in all investigated organs, frequencies of CD4^+^CD25^+^FoxP3^+^ T_REG_ were decreased as compared with the CyA group, but showed increased *ex vivo* proliferation as displayed by their Ki67 expression (Figure 8A). No differences in T_REG_ frequencies were detected by immunohistology (Figure 8B). Contrarily, as compared with the CyA group, intragraft CD8^+^CD122^+^ were clearly increased when NK cells were initially depleted in the early post-transplantation period, but displayed a reduced proliferation profile, although not significant (Figure 8C).

## Discussion

We recently summarized the various facets in which NK cells are involved in SOT and particularly emphasized their relevance for chronic rejection, which may result from NK cells escaping from conventional immunosuppressive regimens (26). However, controversies in the literature make comprehensive studies necessary to substantiate this hypothesis. Conclusions concerning the influence of CyA on NK cell numbers *in vivo* have been specifically drawn from the analysis of peripheral blood cells derived from patients, whereas data on NK cells residing in other compartments including solid organ transplants remain elusive. For instance, Neudoerfl et al. analyzed peripheral blood NK cells from kidney recipients, illustrating that CyA treatment did not affect the percentage of NK cells in the lymphocyte population among KTX patients by comparison to healthy individuals (27).

In our first set of experiments, recipient treatment with CyA significantly reduced intragraft bulk CD3^+^ and CD8^+^ but not CD4^+^ T cell frequencies, whereas no reduction in bulk NK cells was observed for the kidney graft and spleen (Figure 1). Nevertheless, it appeared that distinct NK cell subsets were influenced by CyA, as we detected lower frequencies of intragraft and intrasplenic immature CD27^+^CD11b^−^ NK cells than in untreated controls (Figure 2A). More interestingly, the significant decrease in NKG2D^+^NKp46^+^ NK cell frequencies was obvious in all three investigated organs (Figures 2B–D). The inhibition of NKG2A and a reduction in NKG2D expression have been demonstrated solely for the immunosuppressants mycophenolic acid and rapamycin (18, 28), but it was recently demonstrated that IFNγ production is reduced for NK cells cultured *in vitro* in the presence of high concentrations of CyA (27). We therefore analyzed whether this also applies for NK cells isolated from mice treated with clinically relevant doses of CyA. Importantly, CyA treatment *in vivo* clearly affected IFNγ production of T cells, but not of NK cells. Moreover, the degranulation capacity of both T and NK cells remained unaffected (Figure 3A). Our data therefore stand in contrast to recent *in vitro* studies reporting that treatment of NK cells with cyclosporine and tacrolimus (FK506) resulted in inhibition of both degranulation and IFNγ production (27, 29). Intragraft inflammation of CyA-treated animals was significantly reduced at the mRNA level, which was also observed for IFNγ (Figure 3B). We assume that the decreased IFNγ expression is associated mainly with reduced graft-infiltrating CD8^+^ T cells and their reduced capacity of IFNγ production in the CyA group, as neither NK cell frequencies nor their capacity to produce IFNγ were affected by CyA (Figures 1, 3A). Interestingly, it has been demonstrated that IFNγ neutralization itself does not alter the kinetics of renal allograft rejection (30). However, our data are in line with *in vivo* data demonstrating that NK cell cytotoxicity under CyA treatment is preserved after the transplantation of multipotent adult germline stem cells (maGSCs) into the heart of RAG2^−/−^ and C57BL/6 mice (31).

Having observed that CyA does not impair NK cell frequencies or function, we hypothesized in a second set of experiments that an additional selective targeting of these cells might beneficially affect kidney graft function. Whereas, CyA treatment alone resulted in an improvement of tissue damage compared with the untreated group, this was not further ameliorated by additional NK cell depletion. In contrast, the CyA+NK group demonstrated a significant improvement in graft function (urea, creatinine) as compared to untreated controls (Figure 5). However, additional NK cell depletion reversed the effect of decreased frequencies of antigen-presenting cells in the spleen, but not in the graft or lymph nodes (Figure 4). We can only speculate whether donor-derived APC killing by recipient-derived NK cells accounts for this observation as we could not distinguish between donor and recipient cells in our model (12, 32). Moreover, this would not explain the lack of differences for APCs in graft and lymph nodes between CyA and CyA+NK cell-depleted recipients (Figure 4).

However, by conducting long-term survival experiments until POD56, graft survival was significantly improved in recipients treated with CyA+NK cell depletion as compared with untreated recipients, whereas CyA treatment alone was not sufficient to significantly prolong allograft survival (Figure 7A). Despite the presence of activated intragraft CD4^+^ T_REG_ and higher frequencies of CD8^+^ T_REG_ in the CyA+NK group, we were not able to detect a significant improvement in graft histology (Table 1). With respect to CD4^+^ T_REG_, the presence of FoxP3^+^ cells has already been comprehensively demonstrated in the murine KTX model (33, 34), but their proliferative capacity was never addressed. Speculating on the fact that T_REG_ proliferate in response to antigenic stimuli *in vivo* (35) and that these cells illustrate a greater suppressive capacity *in vitro* (36), we hypothesize that the prolonged long-term survival in the CyA+NK group does not rely solely on the presence of CD4^+^ T_REG_, but instead on the frequency of antigen-specific and proliferating CD4^+^ T_REG_. Although this result is not statistically significant, the induction of CD4^+^FoxP3^+^Ki67^+^ T cells in the graft, spleen and lymph nodes was obvious in the CyA+NK group as compared with the two other groups. Intriguingly, NK cell depletion resulted in a clear induction of intragraft CD8^+^CD122^+^ Treg, a subset, which has been shown to be more potent in suppression of allograft rejection than their CD4^+^CD25^+^ counterparts (37). As numbers of surviving animals was low, both the observation of increased frequencies of proliferating CD4^+^ T_REG_ as well as the induction of CD8^+^ T_REG_ and its relation to NK cell depletion need to be confirmed in independent follow-up studies.

Currently, NK cell transcripts have been associated with antibody-mediated rejection of renal allografts (13, 14). Interestingly, a decrease in the antibody response in the short-term course was not detected for CyA treatment only, but associated mainly with CyA+NK treatment (Figure 6), thus suggesting a positive influence of NK cell depletion on *de novo* antibody generation. We have to admit that the number of animals analyzed in this experiment is small, but the obtained data suggest that the influence of CyA on antibody production needs to be carefully revisited, as CNI agents should suppress the humoral immune response by interfering with T-helper cell signaling (38). Nevertheless, the induction of *de novo* antibodies despite a CyA regimen is still a frequent observation in clinical kidney transplantation (39).

We show for the first time that NK cell depletion combined with CyA synergistically improves renal allograft function, suggesting that selective NK cell targeting might constitute a novel approach to ameliorating outcomes after KTX in the long term. Nonetheless, we are aware of the fact that our study has limitations. First, we did not perform a kinetic analysis of NK cell phenotype and function in CyA-treated recipients post-kidney transplantation. Therefore, we cannot rule out that an earlier or later time point would have brought alternative results. Second, the time point and dosage of NK cell depletion were chosen on the basis of previous studies, where it was observed that application of neutralizing NK1.1 antibody administered daily for seven days resulted in signs of opportunistic infections (data not shown). We therefore decided to apply only a double dose of NK cell-depleting antibody as this was sufficient to efficiently deplete NK cells (Supplemental Figure 4). However, data from infection models suggest that the time point at which NK cells are depleted greatly influences the anti-viral immune response. For instance, delayed NK cell depletion improves the control of persistent LCMV infection more efficiently than does NK cell depletion at day 4 or 5 *post infectionem* (40). Consequently, alternative protocols of NK cell depletion combined with additional immunosuppressants need to be studied in order to gain more insights into the question whether modulation of NK cell response will improve allograft function post-kidney transplantation.

## Data Availability Statement

All datasets generated for this study are included in the manuscript/Supplementary Files.

## Ethics Statement

The animal study was reviewed and approved by Landesamt für Gesundheit und Soziales, Berlin, Germany.

## Author Contributions

MA, TR, KKo, and SS were responsible for experiment design and acquisition and analysis and interpretation of data. ASat, AK, EH, ASar, HR, MS, and KKl assisted with experiments and data analyses. FA provided experimental resources. KKo and TR were responsible for writing of the manuscript. All authors reviewed the manuscript before submission.

### Conflict of Interest

The authors declare that the research was conducted in the absence of any commercial or financial relationships that could be construed as a potential conflict of interest.
