# Supplementary material for: Natural Killer Cells Promote Kidney Graft Rejection Independently of Cyclosporine A Therapy
Source: Front Immunol. 2019 Sep 24;10:2279. doi: 10.3389/fimmu.2019.02279 (PMC6769038; doi:10.3389/fimmu.2019.02279)
Supplement: Supplementary file 1 [file Data_Sheet_1.PDF]

**Supplementary Table 1**: List of antibodies used for flow cytometry

| **Antibody** | **Clone** | **Fluorochrome** | **Company** |
|---|---|---|---|
| CD3 | REA641 | PerCP-Vio700 | Miltenyi |
| CD4 | RM4-5 | BV711 | Biolegend |
| CD8 | 53-6.7 | BV605 | Biolegend |
| NKP46 | 29A1.4 | FITC | eBioscience |
| B220 | RA3-6B2 | APC Fire750 (APC-Cy7) | Biolegend |
| B220 | RA3-6B2 | BV510 | Biolegend |
| CD11c | N418 | PE | Biolegend |
| CD11b | M1/70 | BV785 | Biolegend |
| MHCII | M5/114.15.2 | BV421 | Biolegend |
| CD45 | 30-F11 | UV395 | BD |
| CD27 | M1/70 | Pe-Cy7 | Biolegend |
| NKG2D | CX5 | PE | Biolegend |
| NKG2A | 16A11 | APC | Biolegend |
| CD44 | IM7 | APC-eFluor 780 | eBioscience |
| CD62L | MEL-14 | BV421 | Biolegend |
| KLRG1 | 2F1/KLRG1 | PE-Dazzle594 | Biolegend |
| CD103 | 2E7 | PE | Biolegend |
| CD69 | H1.2F3 | PE-Cy7 | Biolegend |
| CD25 | PC61 | PE | Biolegend |
| FoxP3 | MF-14 | AF 647 | Biolegend |
| Ki67 | SolA15 | Pe-Cy7 | eBioscience |
| CD122 | TM-β1 | FITC | eBioscience |
| CD107a | 1D4B | PE | Biolegend |
| IFNg | XMG1.2 | BV650 | Biolegend |
| Fixable viability dye | | BV510 | Biolegend |

**Supplementary Table 2**: List of Assays on Demand for Real-time RT-PCR

| Gene | Clone | Company |
|---|---|---|
| Granzyme B | Mm00442837_m1 (Lot: 1647982) | Thermo Fisher |
| CXCL 10 | Mm00445235_m1 (Lot: 1662887) | Thermo Fisher |
| IL-1b | Mm00434228_m1 (Lot. 1649751) | Thermo Fisher |
| TNFa | Mm00443528_m1 (Lot:1684087) | Thermo Fisher |
| IFNg | Mm01168134_m1 (Lot: 1667690) | Thermo Fisher |
| Perforin | Mm00812512_m1 (Lot: 1656030) | Thermo Fisher |
