# Supplementary figures and images for: Natural Killer Cells Promote Kidney Graft Rejection Independently of Cyclosporine A Therapy
Source: Front Immunol. 2019 Sep 24;10:2279. doi: 10.3389/fimmu.2019.02279 (PMC6769038; doi:10.3389/fimmu.2019.02279)

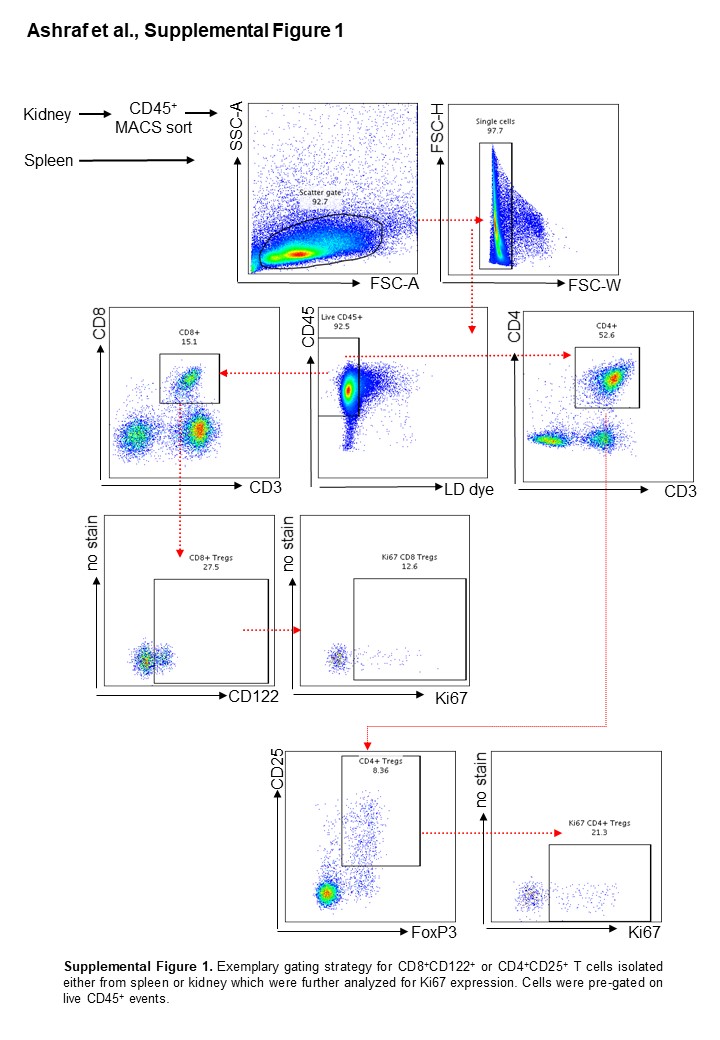

Supplement: Supplementary file 2 [file Image_1.JPEG]

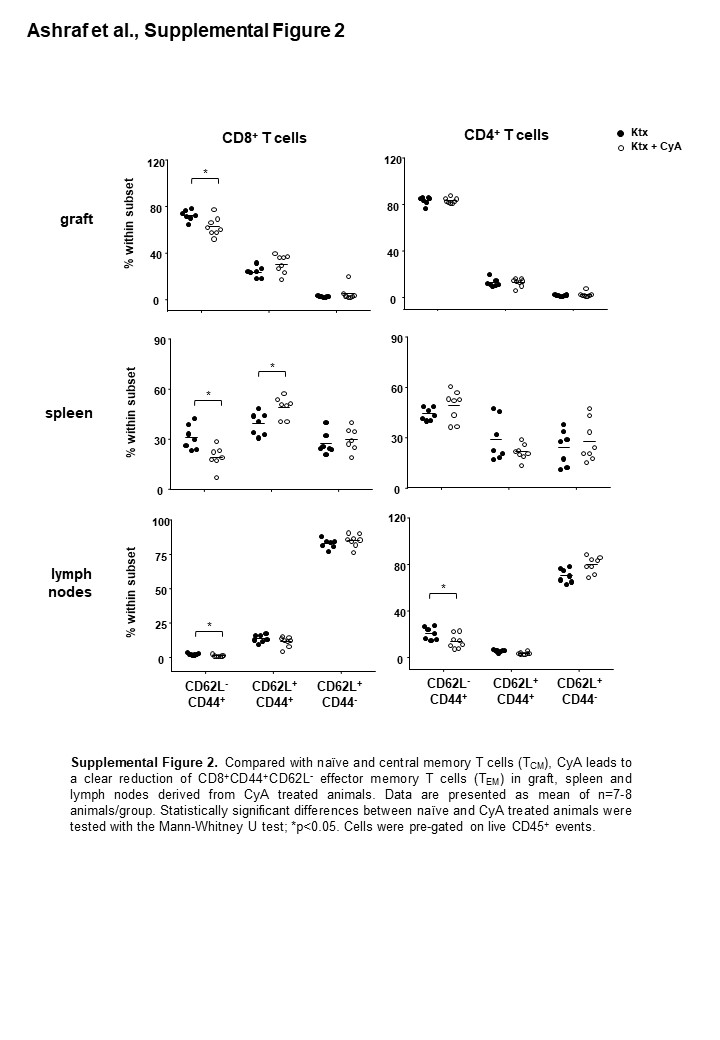

Supplement: Supplementary file 3 [file Image_2.JPEG]

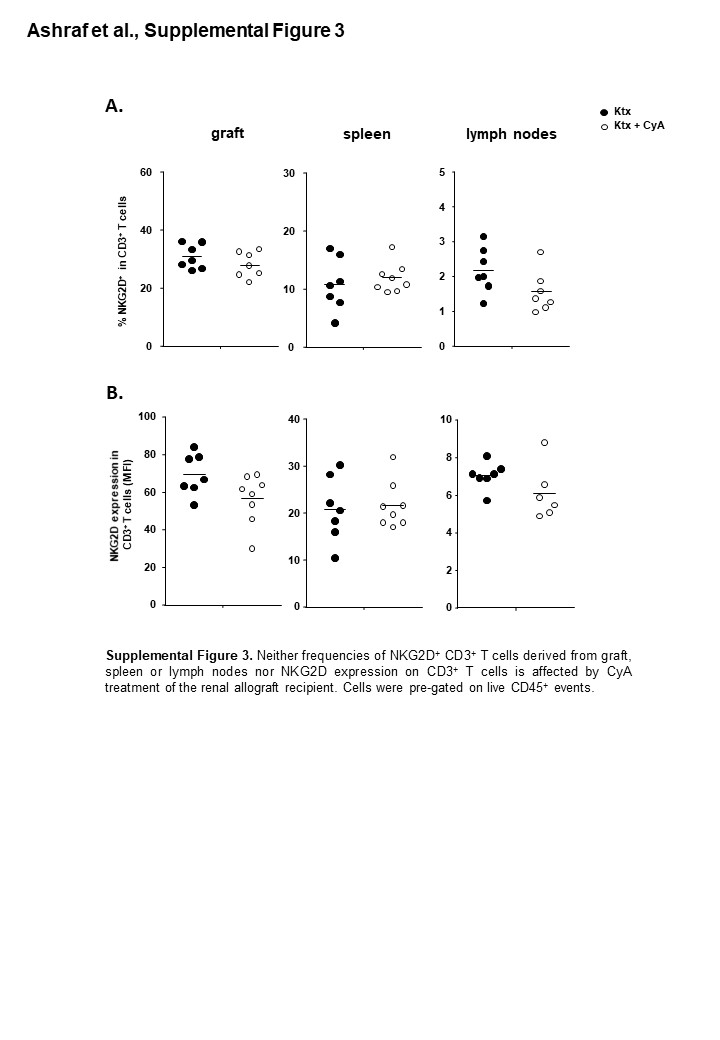

Supplement: Supplementary file 4 [file Image_3.JPEG]

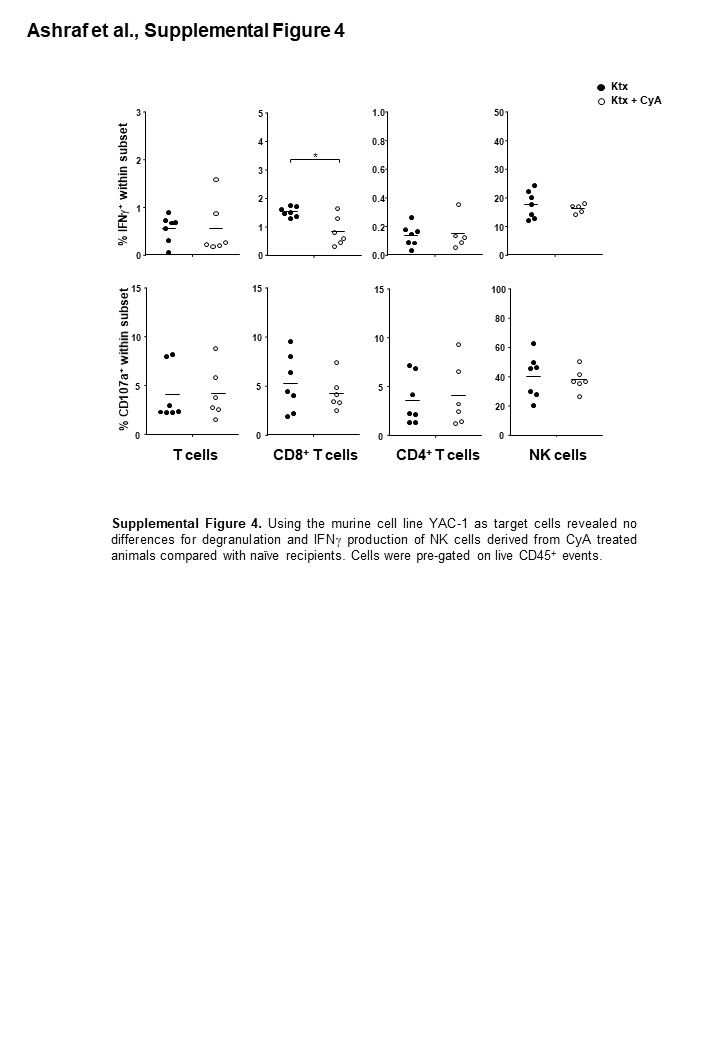

Supplement: Supplementary file 5 [file Image_4.JPEG]

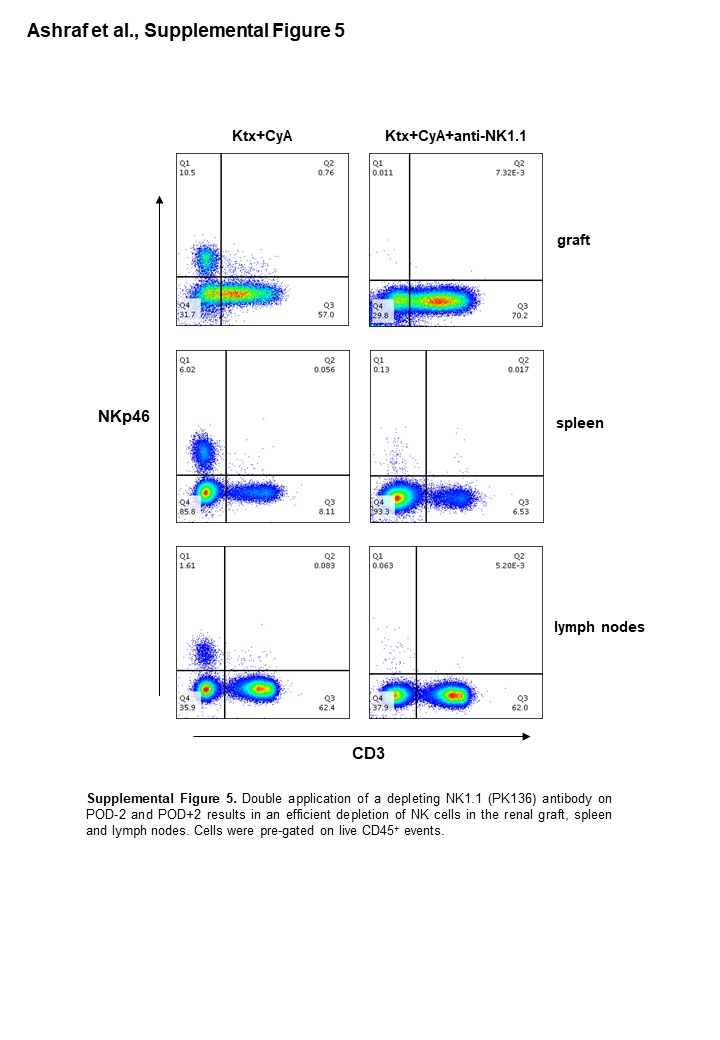

Supplement: Supplementary file 6 [file Image_5.JPEG]
